# Glass-forming ability of La_2_O_3_–Nb_2_O_5_ evaluated via thermophysical properties under microgravity

**DOI:** 10.1038/s41526-025-00520-w

**Published:** 2025-08-25

**Authors:** Atsunobu Masuno, Chihiro Koyama, Shinji Kohara, Shunta Sasaki, Satoshi Izumi, Tomoharu Matsuya, Yuki Mikami, Kenta Yoshida, Hirotaka Kobayashi, Yuki Watanabe, Akitoshi Mizuno, Hirohisa Oda, Yuta Shuseki, Manabu Watanabe, Junpei T. Okada, Takehiko Ishikawa

**Affiliations:** 1 Graduate School of Engineering, Kyoto University, Kyotodaigaku-Katsura, Nishikyo-ku, Kyoto, 615-8520 Japan; 2 Graduate School of Science and Technology, Hirosaki University, 3 Bunkyo-cho, Hirosaki, Aomori 036-8505 Japan; 3 Center for Basic Research on Materials, National Institute for Materials Science (NIMS), 1-2-1 Sengen, Tsukuba, Ibaraki 305-0047 Japan; 4 Human Spaceflight Technology Directorate, Japan Exploration Agency, 2-1-1 Sengen, Tsukuba, Ibaraki 305-8505 Japan; 5 Advanced Engineering Services Co., Ltd., 1-6-1 Takezono, Tsukuba, Ibaraki 305-0032 Japan; 6 National Institute of Technology, Hakodate College, 14-1 Tokura-cho, Hakodate, Hokkaido 042-8501 Japan; 7 Institute for Materials Research, Tohoku University, 2-1-1 Katahira, Aoba-ku, Sendai, Miyagi 980-8577 Japan; 8 Institute of Space and Astronautical Science, Japan Aerospace Exploration Agency, 2-1-1 Sengen, Tsukuba, Ibaraki 305-8505 Japan

**Keywords:** Materials science, Chemical physics, Optical materials, Chemical physics, Glasses

## Abstract

The La_2_O_3_–Nb_2_O_5_ binary system is a unique glass-forming system without conventional network former oxides, exhibiting two distinct glass-forming regions: La_2_O_3_-rich and Nb_2_O_5_-rich compositions. To evaluate its glass-forming ability, the temperature dependence of density, viscosity, and surface tension was measured using the electrostatic levitation furnace aboard the International Space Station (ISS–ELF). Melt density showed linear temperature dependence, and thermal expansion coefficients at 2000 K varied from 2.5 × 10^−5^ to 4.0 × 10^−5^ K^−1^. Substantial undercooling was observed for glass-forming compositions. Viscosity measurements above the melting point revealed that both La_2_O_3_-rich and Nb_2_O_5_-rich melts behave as fragile liquids. Activation energy derived from viscosity data was higher for glass-forming compositions. These results suggest that glass-forming ability can be assessed based on undercooling and activation energy across a wide compositional range, including non-glass-forming melts. The ISS–ELF experiments provide a valuable platform for understanding glass formation in systems inaccessible by terrestrial techniques.

## Introduction

Oxide glass science is based on the concept of three-dimensional random network formation through corner-sharing tetrahedral units of network former oxides (NWFs), such as SiO_2_ and P_2_O_5_^1,2^. Typically, a high concentration of NWFs is required for glass formation, limiting the range of chemical compositions explored in glass science. However, recent advancements in levitation techniques have started to overcome these traditional constraints^3–6^. By preventing heterogeneous nucleation at the interface between the melt and its container, these techniques allow for deeper undercooling of melts^7^. As a result, even materials with very low glass-forming ability can solidify into glasses without crystallizing during cooling. In the past two decades, levitation techniques have successfully produced systems such as *A*O–SiO_2_ and *R*_2_O_3_–B_2_O_3_ glasses containing minimal amounts of NWFs, where *A* denotes alkaline earth metals and *R* denotes rare-earth elements or Y^4,8–12^. Additionally, several glass compositions without NWFs have been developed, including Al_2_O_3_-, TiO_2_-, Nb_2_O_5_-, WO_3_-, MoO_3_-, Ga_2_O_3_-, and Ta_2_O_5_-based binary glasses^13–22^. These unconventional glasses often exhibit exceptional physical properties, which are attributed to their densely packed glass structures that lack conventional tetrahedral networks^23,24^. This emerging class of glasses is expanding the horizons of glass science, paving the way for new possibilities in both fundamental research and practical applications^25,26^.

Despite these advancements, the mechanisms behind unconventional glass formation without three-dimensional networks are still not well understood. Because glass forms by cooling from a melt, it is essential to gather temperature-dependent thermophysical data—such as density, viscosity, and surface tension—across a broad temperature range, from above the melting point to the supercooled state. Levitation techniques enable access to this wide temperature range and allow for accurate measurements of thermophysical properties^27,28^. Among the various levitation methods, the electrostatic levitation furnace (ELF) is particularly advantageous for accurate measurements because it maintains the levitated melt in an almost perfectly spherical shape^29^. The ELF has been widely applied to metals and provided valuable thermophysical data^30^. However, levitating oxide materials on Earth presents considerable challenges because achieving the high electric field necessary to counteract gravity requires a high vacuum environment, which in turn leads to considerable evaporation from oxide melts^31,32^. Oxide samples can be levitated under a pressurized environment without evaporation, but it is very hard to maintain the sample charge at high temperature for stable levitation. Only a few oxide samples have been successfully levitated, melted to obtain their thermophysical properties in the pressurized ELFs^33–35^. To overcome these challenges, advancements in ELF technology have been made, including performing experiments in space. Notably, the ELF installed on the Japanese Experiment Module “Kibo” aboard the International Space Station (ISS) allows for the measurement of thermophysical properties of oxide melts under conditions close to atmospheric pressure^36,37^.

The thermophysical properties of oxide melts are combined with their atomic structural data measured by X-ray and neutron diffraction experiments on the ground, as well as theoretical approaches for a better understanding of their glass-forming abilities^6,38,39^. These investigations have offered valuable insights into the structural characteristics of non-glass-forming melts at high temperatures, such as ZrO_2_, Al_2_O_3_, UO_2_, and *R*_2_O_3_^40–44^.

**Fig. 1:**
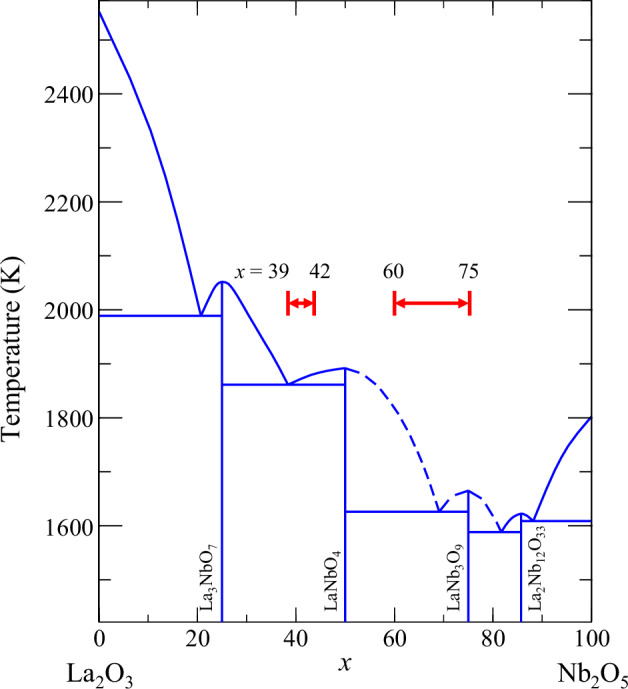
Phase diagram of the La_2_O_3_–Nb_2_O_5_ binary system^45,48^. Red arrows indicate glass-forming regions observed using the aerodynamic levitation technique.

In this study, we investigate the La_2_O_3_–Nb_2_O_5_ binary system. Using an aerodynamic levitation furnace, we found that certain compositions produced colorless, transparent glasses with exceptionally high refractive indices and low wavelength dispersion, even in the absence of NWFs^18,45,46^. Notably, the glass-forming region is divided into two distinct areas: a La_2_O_3_-rich region (39–42 mol% Nb_2_O_5_) and a Nb_2_O_5_-rich region (60–75 mol% Nb_2_O_5_), with a non-vitrifying composition range separating them^45,47,48^. This indicates that the primary components driving glass formation change depending on the composition. Figure 1 shows the phase diagram of the binary system, highlighting two distinct glass-forming regions. The thermal properties, including glass transition temperatures, show considerable variation between these regions. Detailed structural analyses revealed marked changes in connectivity across these regions^47^. Consequently, we propose that the melt properties contain key indicators for understanding the distinct glass-forming abilities and non-vitrifying behaviors in the La_2_O_3_–Nb_2_O_5_ system. This study thoroughly investigates the chemical composition range of La_2_O_3_–Nb_2_O_5_ melts to better understand these phenomena.

## Results

### Density and undercooling temperature

**Fig. 2:**
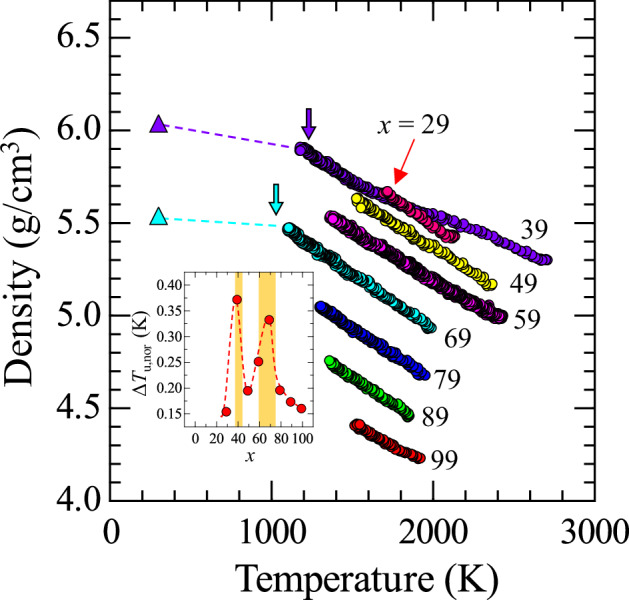
Temperature dependence of the density of (99−*x*)La_2_O_3_–*x*Nb_2_O_5_–1Fe_2_O_3_ melts (*x* = 29, 39, 49, 59, 69, 79, 89, and 99). The downward arrows and triangles represent the glass transition temperature and the density of 30La_2_O_3_–70Nb_2_O_5_ and 60La_2_O_3_–40Nb_2_O_5_ glasses, corresponding to *x* = 69 and 39, respectively. The relative error is estimated to be 2.5% (not shown)^37^. The inset depicts the composition dependence of Δ*T*_u,nor_, with yellow regions indicating the glass-forming regions. The dashed line serves as a guide to the eye.

Figure 2 shows the temperature dependence of density for (99−*x*)La_2_O_3_–*x*Nb_2_O_5_–1Fe_2_O_3_ (*x* = 29, 39, 49, 59, 69, 79, 89, and 99) melts. Data were typically collected in the temperature range of 1500–2000 K, while compositions with higher melting points provided data up to 2500 K. For *x* = 39, a bending behavior was observed at approximately 2000 K, whereas the other compositions showed a linear relationship between density and temperature. It is worth noting that the bending temperature for *x* = 39 is close to the liquidus temperature of 1868 K. From the linear fit to the data, an approximate formula for the temperature dependence of melt density was obtained as *ρ*(*T*) = *ρ*(*T*_L_) + (d*ρ*(*T*)/d*T*)·(*T* − *T*_L_) where *ρ*(*T*_L_) is the density at the liquidus temperature *T*_L_. Table 1 summarizes the parameters of the equation. There was no considerable composition dependence observed for the temperature coefficient *dρ*(*T*)/*dT*. As *x* increased, density decreased monotonically at a given temperature, reflecting the substitution of the heavier molecular weight La_2_O_3_ with Nb_2_O_5_.

**Table 1:** Parameters for the density equation *ρ*(*T*) = *ρ*(*T*_L_) + (d*ρ*(*T*)/d*T*)·(*T* − *T*_L_) for (99−*x*)La_2_O_3_–*x*Nb_2_O_5_–1Fe_2_O_3_ melts

*x*	*ρ*(*T*_L_) (g cm^−3^)	d*ρ*(*T*)/d*T* (10^−4^ g cm^−3^ K^−1^)	*T*_L_ (K)
29	5.488	−5.9	2002
39	5.552	−5.0	1868
49	5.424	−5.4	1893
59	5.289	−5.2	1815
69	5.137	−6.0	1644
79	4.868	−5.7	1613
89	4.584	−5.7	1642
99	4.273	−4.6	1803

The lowest temperature measured for each composition corresponds to the crystallization temperature from the melt, except for those in the glass-forming regions. Some of the recovered samples exhibited a white appearance, indicating the evaporation or reduction of Fe during levitation. No significant differences were observed in the physical properties between samples that retained Fe and those from which Fe was depleted. Therefore, the impact of Fe_2_O_3_ on the physical properties in the present system is considered negligible. Accordingly, the liquidus temperatures (*T*_L_) of the melts were referenced from the La_2_O_3_–Nb_2_O_5_ binary phase diagram, as shown in Fig. 1. The maximum undercooling temperature (Δ*T*_u_) was determined as the difference between the liquidus temperature (*T*_L_) and the lowest temperature (*T*_lw_) reached before crystallization. A smaller Δ*T*_u_ indicates a higher tendency for crystallization. The reduced undercooling temperature Δ*T*_u,nor_ is defined as Δ*T*_u,nor_ = Δ*T*_u_/*T*_L_. The inset of Fig. 2 shows the composition dependence of Δ*T*_u,nor_, highlighting two peaks at *x* = 39 and 69, which correspond to compositions capable of glass formation. Additionally, Δ*T*_u,nor_ values for *x* = 49 and 59 were higher than those for *x* = 29, 79, 89, and 99 compositions. This suggests that the *x* = 49 and 59 melts have a greater potential for glass formation compared to the *x* = 29, 79, 89, and 99 melts. Notably, a sample with *x* = 49 formed glass in space, even though this composition lies outside the glass-forming regions on the ground. Unlike levitation using an ADL, where the melt often experiences violent rotation owing to gas flow, levitation in the ISS–ELF is highly stabilized, enabling compositions at the boundary of non-vitrification to potentially form glass. In glass science, the temperature difference (Δ*T*) between the glass transition temperature and the crystallization temperature, typically measured using thermal analysis methods such as DSC or DTA, serves as an indicator of glass stability against crystallization. The Δ*T*_u,nor_ determined through a levitation technique provides a more intrinsic and reliable measure for assessing glass-forming ability. However, this remains an experimental observation and does not yet reach the level of a comprehensive theoretical framework that integrates historical metrics of glass-forming ability.

Downward arrows in Fig. 2 indicate the glass transition temperature (*T*_g_) of 60La_2_O_3_–40Nb_2_O_5_ and 30La_2_O_3_–70Nb_2_O_5_ glasses, corresponding to *x* = 39 and 69 melts, respectively. A distinct bend in the temperature dependence of density, indicative of the glass transition, was frequently observed for the glass-forming liquids in the ISS–ELF experiments^49^. At *x* = 39, however, the data approached *T*_g_, but no distinct bending of the density curve was observed. For both *x* = 39 and *x* = 69, density data for the corresponding glasses at room temperature are available and are plotted as triangles in the figure. The dashed lines connecting the points of the glass transition temperature and room temperature exhibit a gentler slope compared to the high-temperature melt data. This behavior is consistent with expectations, as the thermal expansion coefficient of solids is typically smaller than that of liquids.

### Thermal expansion coefficient

**Fig. 3:**
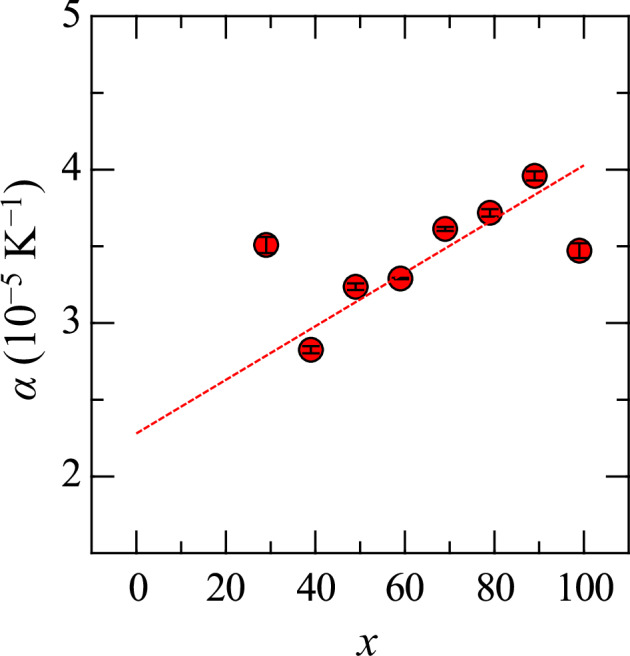
Linear thermal expansion coefficient α at 2000 K for (99−*x*)La_2_O_3_–*x*Nb_2_O_5_–1Fe_2_O_3_ melts. The dashed line represents the fitted trend.

The thermal expansion coefficient (*β*) was determined from the volume data. First, the temperature dependence of the volume was fitted to a linear equation: *V*(*T*) = A*T* + B, where A and B are constants. The thermal expansion coefficient *β*_2000_ at 2000 K was then calculated using Eq. (1), where *V*_2000_ represents the volume at 2000 K. The linear thermal expansion coefficient (*α*) was subsequently derived as *α*_2000_ = (1/3) *β*_2000_. These parameters are summarized in Table 2. Previous studies^50–52^ reported *β* values at its melting point of 2.23 (Al_2_O_3_), 5.35 (Y_2_O_3_), 4.5 ± 0.5 (Gd_2_O_3_), 3.7 ± 0.4 (Tb_2_O_3_), 4.6 ± 0.4 (Ho_2_O_3_), 10.0 ± 0.8 (Er_2_O_3_), 2.2 ± 0.6 (Tm_2_O_3_), 6.5 ± 0.9 (Yb_2_O_3_) and 5.0 ± 0.9 (Lu_2_O_3_) in units of 10^−5^ K^−1^. The *β*_2000_ values obtained in this study were consistent with those previously reported data. Figure 3 shows the composition dependence of *α*_2000_ for the melts, with values ranging from 2.5 × 10^−5^ to 4.0 × 10^−5^ K^−1^. While some scatter was observed, a linear fit was applied, resulting in the equation *α*(*x*) = (1.75 ± 0.45) × 10^−7^ × *x* + (2.28 ± 0.28) × 10^−5^. From the extrapolation of the fitted line, the linear thermal expansion coefficients at 2000 K of La_2_O_3_ and Nb_2_O_5_ were estimated as α_La2O3_ = (2.28 ± 0.28) × 10^−5^ K^−1^ and α_Nb2O5_ = (4.03 ± 0.73) × 10^−5^ K^−1^, respectively.

1 $${\beta }_{2000}=\frac{1}{{V}_{2000}}\frac{{\rm{d}}V}{{\rm{d}}T}$$

**Table 2:** Thermal expansion coefficient parameters for (99−*x*)La_2_O_3_–*x*Nb_2_O_5_–1Fe_2_O_3_ melts

*x*	*A* (10^−4^ mm^3^ K^−1^)	*B* (mm^3^)	*V*_2000_ (mm^3^)	*β*_2000_ (10^−5^ K^−1^)	*α*_2000_ (10^−5^ K^−1^)
29	3.73	2.79	3.54	10.5	3.51
39	2.44	2.39	2.87	8.48	2.83
49	3.84	3.19	3.96	9.71	3.24
59	3.29	2.68	3.33	9.87	3.29
69	4.82	3.48	4.45	10.8	3.61
79	3.34	2.33	3.00	11.2	3.72
89	4.38	2.81	3.69	11.9	3.96
99	3.53	2.68	3.39	10.4	3.47

### Viscosity

**Fig. 4:**
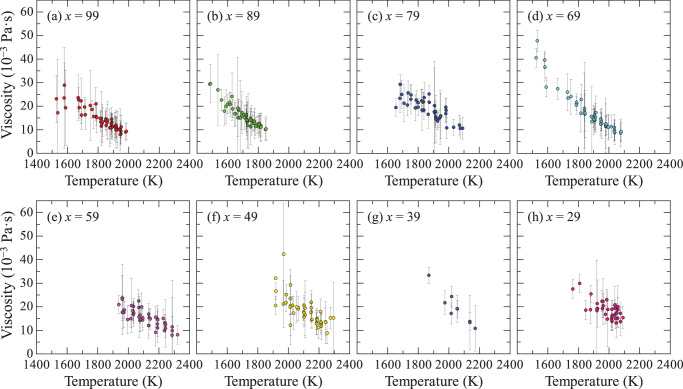
Temperature dependence of viscosity for (99−x)La_2_O_3_–xNb_2_O_5_–1Fe_2_O_3_ melts. **a** *x* = 99. **b** *x* = 89. **c** *x* = 79. **d** *x* = 69. **e** *x* = 59. **f** *x* = 49. **g** *x* = 39. **h** *x* = 29.

Figure 4 shows the temperature dependence of viscosity for (99−*x*)La_2_O_3_–*x*Nb_2_O_5_–1Fe_2_O_3_ (*x* = 29, 39, 49, 59, 69, 79, 89, and 99) melts. Viscosity measurements were performed using the drop oscillation method with the ISS–ELF, which has a measurable viscosity range of 10^−1^–10^−3^ Pa s. As a result, viscosity data were collected at temperatures considerably higher than both the glass transition and crystallization temperatures. Although some compositions had a limited number of data points, the results consistently indicated that viscosity decreased as temperature increased.

### Surface tension

**Fig. 5:**
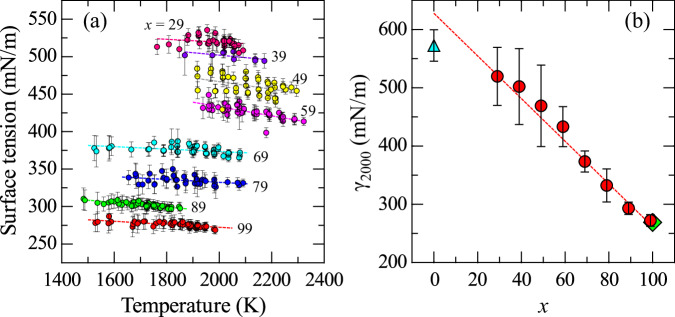
Surface tension for (99−*x*)La_2_O_3_–*x*Nb_2_O_5_–1Fe_2_O_3_ melts. **a** Temperature dependence of the surface tension. **b** Composition dependence of the surface tension at 2000 K. The dashed line represents a linear fit. The surface tension values for La_2_O_3_ and Nb_2_O_5_ are shown as a triangle and a diamond, respectively, for refs. ^54,55^.

Figure 5 summarizes the temperature dependence of the surface tension for (99−*x*)La_2_O_3_–*x*Nb_2_O_5_–1Fe_2_O_3_ melts. The surface tension of these melts shows a mild linear temperature dependence, as depicted in Fig. 5a. As temperature increases, the surface tension decreases. The values range from 250 to 550 mN/m, which are consistent with previously reported data for glass-forming oxide liquids^53^. Figure 5b shows the composition dependence of surface tension at 2000 K, clearly showing that the surface tension decreases monotonically as the Nb_2_O_5_ content increases. The dashed line represents a linear fit to the equation γ_2000_(*x*) = (−3.66 ± 0.23)·*x* + (627 ± 21). Based on this equation, γ_2000_La2O3_ and γ_2000_Nb2O5_ are estimated to be 627 ± 21 and 262 ± 46 mN/m, respectively. The surface tension of La_2_O_3_ at the melting point under 1 atm was obtained as 572.5 ± 27 mN/m by using a pendant droplet method^54^. The surface tension of Nb_2_O_5_ was determined to be 269 mN/m at 1934 K by using the maximum bubble pressure method^55^. Both values obtained in this study are consistent with previously reported data^54,55^. The plotted data in Fig. 5(b) show a slight deviation from the linear fit, potentially indicating the presence of excess enthalpy. However, owing to the limited data, confirming nonlinearity remains challenging.

## Discussion

**Fig. 6:**
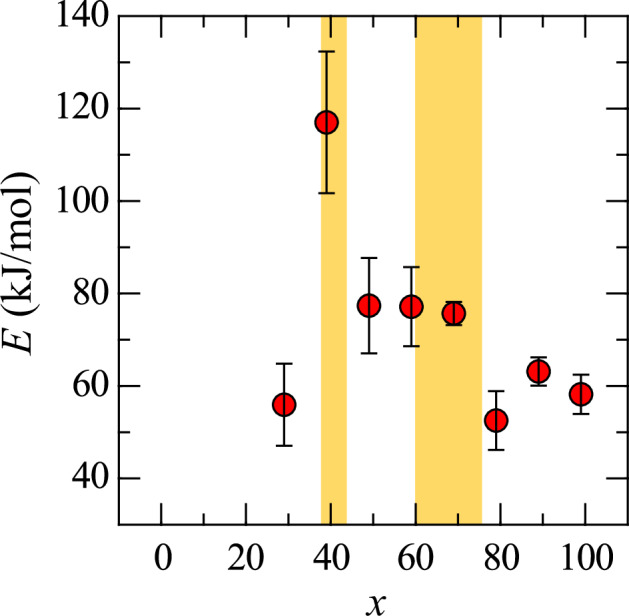
Composition dependence of *E* for (99−*x*)La_2_O_3_–*x*Nb_2_O_5_–1Fe_2_O_3_ melts. Yellow regions indicate glass-forming regions.

To analyze the viscosity data, the Andrade equation (Eq. (2)) was used, where *η* is the viscosity, *D* is the pre-exponential factor, *E* is the activation energy, *R* is the gas constant, and *T* is the temperature. A linear fit of the ln *η* data based on the Andrade equation allowed for the determination of *E*. Figure 6 shows the composition dependence of *E*, with yellow regions indicating the glass-forming regions. The results show that the activation energy for *x* = 39 was considerably higher than for other compositions. Similarly, *E* for *x* = 49, 59, and 69 was also higher compared to *x* = 29, 79, 89, and 99. This trend aligns with the glass-forming region, where higher *E* values indicate an enhanced glass-forming ability. A lower *E* suggests that ions (or flow units) can move more freely, promoting viscous flow, which correlates with a reduced glass-forming ability and an increased tendency for crystallization. The higher *E* indicates restricted ion mobility, preventing the atomic structure from easily transitioning to a crystalline state. Instead, the structure remains “frozen” in a liquid-like state, which supports glass formation during cooling. The glass-forming ability inferred from the viscosity data is consistent with the trends observed in the Δ*T*_u,nor_ values obtained from the cooling curves. This consistency confirms the relationship between high activation energy, glass-forming ability, and the crystallization resistance of the melts.

2 $$\eta =D\exp \left(\frac{E}{{RT}}\right)$$

**Fig. 7:**
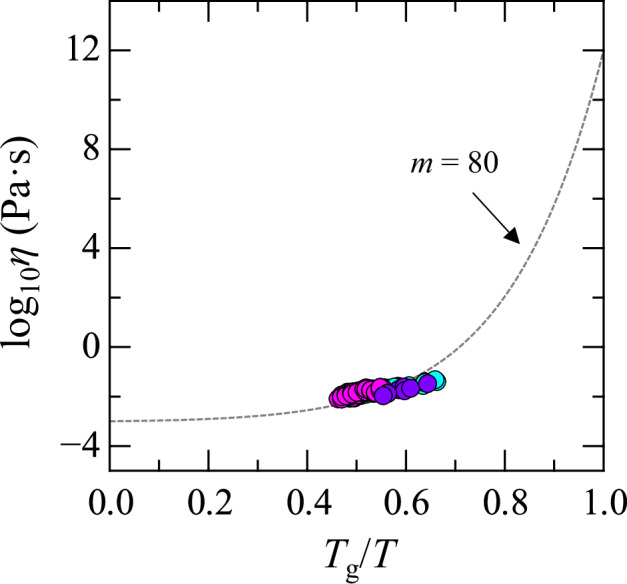
Angell plot for (99−*x*)La_2_O_3_–*x*Nb_2_O_5_–1Fe_2_O_3_ melts (violet: *x* = 39, magenta: *x* = 59, cyan: *x* = 69). The dotted curve represents the calculation using the simplified MYEGA equation with *m* = 80.

The temperature dependence of viscosity is a key parameter for evaluating glass-forming ability, as proposed by Angell^56,57^. Figure 7 shows the Angell plot for *x* = 39, 59, and 69 melts, which lie in the glass-forming regions. Data for other compositions are not shown due to the absence of glass transition temperature. The vertical axis represents the logarithmic viscosity (*η*), while the horizontal axis shows the inverse of temperature, normalized by the glass transition temperature (*T*_g_). The *T*_g_ values were obtained from the literature^45^. The plotted data strongly indicate that all the melts are fragile liquids because they deviate from the linear relationship. An estimation of *m* is made using the Mauro–Yue–Ellison–Gupta–Allan (MYEGA) model (Eq. (3))^58^:

3 $${\log }_{10}\eta \left(T\right)={\log }_{10}{\eta }_{{\infty }}+\left(12-{\log }_{10}{\eta }_{{\infty }}\right)\frac{{T}_{g}}{T}\exp \left[\left(\frac{m}{12-{\log }_{10}{\eta }_{{\infty }}}-1\right)\left(\frac{{T}_{g}}{T}-1\right)\right]$$

where log_10_*η*_∞_ represents the high-temperature limit of viscosity. However, accurately determining the fragility index (*m*) from viscosity data only at higher temperatures is challenging. For rough estimation, the simplified version of the MYEGA equation (Eq. (4))^59^, where log_10_*η*_∞_ is fixed at −3, is often employed:

4 $${\log }_{10}\eta =-3+15\frac{{T}_{g}}{T}\exp \left[\left(\frac{m}{15}-1\right)\left(\frac{{T}_{g}}{T}-1\right)\right]$$

The dashed curve in Fig. 7 was calculated using the equation with *m* = 80. Most of the experimental data align closely with this curve. One notable feature is that the temperature dependence of viscosity in the experimental data appears slightly weaker than that predicted by the simplified MYEGA model. Although the model assumes a sharp increase in viscosity as temperature decreases, it remains unclear whether adjusting only log_10_*η*_∞_ in the original MYEGA formulation is sufficient to capture this behavior. Future work should therefore focus on accurate viscosity measurements at lower temperatures, near the glass transition temperature, using alternative methods.

The density, viscosity, and surface tension of La_2_O_3_–Nb_2_O_5_ binary melts were measured based on their temperature dependence using the ISS–ELF. The compositions studied ranged from *x* = 29 to 99. Density measurements were taken from temperatures above 2300 K down to deeply undercooled states, with the degree of undercooling varying by composition and being most pronounced for glass-forming compositions. The linear thermal expansion coefficient *α*, estimated at 2000 K, was approximately 2.5 × 10^−5^ to 4.0 × 10^−5^ K^−1^. Viscosity measurements were performed at temperatures above the melting point, in the range of approximately 10^−1^–10^−3^ Pa·s. The activation energy was determined using the Andrade equation and was found to be dependent on the composition. Higher activation energy values were found in glass-forming regions, indicating the limited ion mobility that facilitates glass formation. The fragility index (*m*) was estimated using the simplified MYEGA equation, revealing that the melts are highly fragile liquids, with *m* values exceeding 70. Surface tension demonstrated a linear relationship with composition, further supporting the connection between melt properties and glass-forming ability. These findings show that the glass-forming ability can be effectively assessed using thermophysical parameters obtained through the ISS–ELF.

## Methods

### Sample preparation

High-purity powders of La_2_O_3_, Nb_2_O_5_, and Fe_2_O_3_ were stoichiometrically mixed to achieve compositions of (99−*x*)La_2_O_3_–*x*Nb_2_O_5_–1Fe_2_O_3_ (*x* = 0, 9, 19, 29, 39, 49, 59, 69, 79, 89, and 99). Fe_2_O_3_ was added to improve absorption efficiency for the 980-nm semiconductor laser used in the ISS–ELF experiments. The mixtures were pressed into pellets and sintered at 1000 °C for 12 h in air. The sintered pellet was then crushed to prepare target pieces for the aerodynamic levitation (ADL) furnace, which is used to produce spherical ceramics for the ISS–ELF. In the ADL furnace, a piece of the target material was levitated using an O_2_ gas flow. A CO_2_ laser was used to melt the levitated sample for several seconds, and the melt was rapidly cooled to room temperature by turning off the laser. This process solidified the material into spherical ceramics. Depending on their composition in the previously reported glass-forming regions^45^, some compositions formed glasses, while others crystallized. The diameter of the resulting spherical ceramics ranged from 1.6 to 2.1 mm, which is optimized for the ISS–ELF sample holder^36^. Preliminary tests were performed to ensure that the spherical ceramics maintained their shape during rocket transport and storage in space. Samples with a high La_2_O_3_ content (*x* = 0, 9, and 19) were excluded owing to their high deliquescence and fragility. Three samples from each of the remaining compositions (*x* = 29, 39, 49, 59, 69, 79, 89, and 99) were weighed, placed in the sample holders designed for the ISS–ELF, and then transported to the ISS aboard the H-II Transfer Vehicle 7 (HTV7)^60^. Upon arrival, astronauts installed the sample holders in the ISS–ELF. The experiments were remotely controlled by our team from the Tsukuba Space Center of JAXA in Japan^37^.

### Measurement conditions at ISS

The ISS–ELF chamber was filled with dry air, N_2_, or Ar at a pressure of 2 atm. Each sample was pushed from the holder into the center of the chamber using a rod and levitated by an electric field. The sample, carrying positive charges in the range of 10^−11^–10^−12^ C, was stabilized at the center using three pairs of orthogonally arranged electrodes. Its position was monitored by two He–Ne laser position-sensing systems. Voltage adjustments between the electrodes ensured the stable positioning of the sample. The levitated samples were heated by four 40 W, 980 nm diode lasers arranged tetrahedrally for uniform heating. As the temperature increased, the charge on the sample occasionally reversed, leading to instability in levitation. High-speed feedback control was used to maintain stable positioning. Once melted, the oxide samples formed nearly perfect spherical shapes owing to surface tension in the microgravity environment. Magnified images of the samples were captured using ultraviolet backlighting at each temperature^37,61^.

**Fig. 8:**
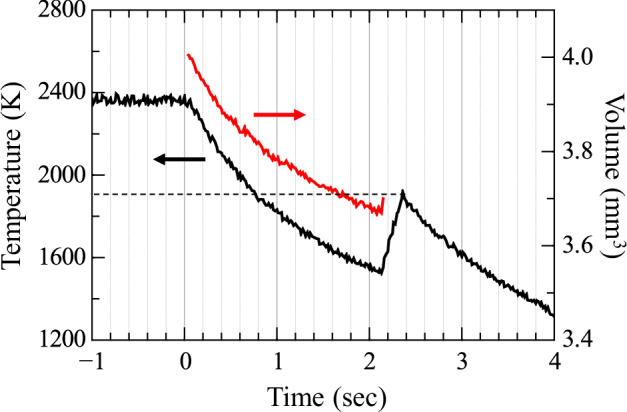
Cooling curve of the 50La_2_O_3_–49Nb_2_O_5_–1Fe_2_O_3_ melt in the ISS-ELF experiments. The time axis begins at the moment the laser is turned off. The dashed line represents the melting point *T*_m_ of 50La_2_O_3_–50Nb_2_O_5_, as determined from the phase diagram^45^.

Temperature measurements were performed using a pyrometer with a wavelength range of 1.45–1.8 μm. After the laser was turned off, the melt temperature decreased quickly below the melting point and then increased to the melting point owing to the release of latent heat during crystallization. The measured temperatures were corrected by adjusting the emissivity values to align the peak temperature after the recalescense with the melting temperature reported in the literature. Figure 8 shows a representative cooling curve obtained in the ISS-ELF experiments for the 50La_2_O_3_–49Nb_2_O_5_–1Fe_2_O_3_ melt, corresponding to a composition that does not vitrify under ground-based conditions. The temperature rapidly increased after crystallization, occurring 2.15 s after the laser was turned off. The dashed line indicates the melting point, approximately 1893 K. The undercooling temperature Δ*T*_u_ reached approximately 400 K, a value that conventional crucible-based melt-quenching methods cannot achieve. This is because the levitated melt is highly stabilized in the ISS-ELF, whereas in an ADL on the ground, the melt is constantly rotated and distorted by the gas flow, which promotes crystallization. While it may be of interest to investigate the effect of gravity on crystallization or glass formation, such comparisons are difficult due to numerous differences in experimental conditions beyond gravity between ISS-ELF and ground-based systems. The ISS–ELF effectively extended the accessible temperature range both above and below the melting point *T*_m_. Thermophysical property data were collected from temperatures below *T*_m_ just before crystallization occurred.

### Density analysis

The density *ρ* of the melts was calculated using the formula *ρ* = *m*_s_/*V*, where *m*_s_ is the mass of the sample and *V* is its volume. The volume *V* was determined by analyzing magnified images taken during the experiments. Before the melt experiments, pixel measurements in the images were calibrated to actual sample dimensions (in mm) using reference images of a levitated stainless-steel ball with a known diameter of 2.0 mm, levitated in the ISS–ELF. While the melts were nearly spherical, slight distortions were observed. To accurately calculate the volume, 400 edge points were identified from the nearly spherical sample image and converted into polar coordinates (*R*, *θ*). These coordinates were fitted to sixth-order spherical harmonic functions using Eq. (5), where *P*_*n*_(cos*θ*) represents the *n*-th order Legendre polynomials and *c*_*n*_ are the coefficients (*n* = 0–6) determined by minimizing the *F* value from Eq. (6). The total volume *V* of the sample was then calculated using Eq. (7). During the cooling process, magnified images of the specimen were captured at approximately 10-K intervals within a few seconds. The time-dependent volume change obtained in the ISS-ELF experiments is shown in Fig. 2. The melt volume decreased monotonically in accordance with thermal expansion. The temperature dependence of density was derived from the data series. The lowest measurable temperature varied depending on the sample composition. The mass (*m*_s_) of each specimen was measured before transport to the ISS. While this value was used for density calculations during the space experiments, it was later corrected upon return to Earth to account for any minor evaporation that may have occurred during high-temperature melting. The uncertainty in the density measurements has been thoroughly discussed in several previous studies and is estimated to be approximately 2–2.5%^37,50,51,62^. In the present work, we adopted an uncertainty of 2.5% based on the prior report^37^.

5 $$R\left(\theta \right)=\mathop{\sum }\limits_{n=0}^{6}{c}_{n}{P}_{n}\left(\cos \theta \right)$$

6 $$F=\mathop{\sum }\limits_{j=1}^{400}{\left\{{R}_{j}-{R}_{j}\left(\theta \right)\right\}}^{2}$$

7 $$V=\frac{2\pi }{3}{\int }_{0}^{\pi }{R}^{3}\left(\theta \right)\,\sin \theta \,{\rm{d}}\theta$$

### Surface tension and viscosity analysis

**Fig. 9:**
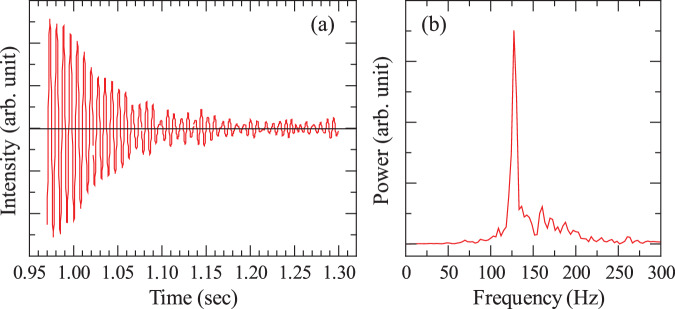
Data obtained by the droplet oscillation method for the 99Nb_2_O_5_–1Fe_2_O_3_ melt. **a** The oscillation signal. **b** The FFT of the oscillation signal.

The surface tension *γ* and viscosity *η* of the melts were measured using the drop oscillation method^37,63^. A collimated laser beam, used to sense the sample’s position, was split by a beam splitter, projecting the sample’s shadow onto a power meter. Sinusoidal voltages were applied to the vertical electrodes to induce oscillatory deformation in the sample. The deformation was detected as fluctuations in the power of the He–Ne laser beam received by the power meter. Sample oscillations began when the excitation voltage frequency neared the mode-2 oscillation frequency *f*_2_ of the sample. After the excitation voltage was removed, the oscillations gradually decayed owing to the viscosity of the melt. Signals from the power meter were recorded starting 1 s before the excitation stopped, with a time resolution of 5000 Hz. Figure 9a shows a typical power meter signal for the 99Nb_2_O_5_–1Fe_2_O_3_ melt. The oscillation decay time τ was extracted from the data as 0.064 s. Figure 9b shows the corresponding Fast Fourier Transform (FFT) result, with the resonance frequency *f*_2_ determined to be 127.83 Hz. Surface tension *γ* and viscosity *η* were calculated using the following equations:

8 $$\gamma =\frac{\rho {r}^{3}{\left(2\pi {f}_{2}\right)}^{2}}{8}$$

9 $$\eta =\frac{\rho {r}^{2}}{5\tau }$$

where *r* is the radius of the melt. For the case shown in Fig. 3, the surface tension *γ* and viscosity *η* were calculated to be 272.6 mN/m and 11.3 mPa s, respectively. During the drop oscillation measurements, sample temperature is kept at a constant temperature. The drop excitation and signal measurements are conducted several times by sweeping the correct resonance frequency. Then, the heating laser powers are adjusted to the next temperature where drop oscillations are conducted. This sequence is repeated with the temperature interval around 20–30 K. These measurements provided accurate evaluations of the thermophysical properties, contributing to a better understanding of melt behavior under microgravity conditions^49,50,64^. The error evaluation for surface tension and viscosity measurements in drop oscillation experiments conducted using the ISS–ELF has been thoroughly discussed in previous studies^37^. In the present work, the same error estimation methodology was applied.

## Data Availability

The authors declare that the data supporting the findings of this study are available within the paper. Should any raw data files be needed in another format they are available from the corresponding author upon reasonable request.
